# Discovering the Diversity and Evolution of Danascelinae: A New Genus and Species from Eastern Asia and Insights into the Phylogenetic Placement of This Subfamily in Endomychidae (Coleoptera) †

**DOI:** 10.3390/insects16111178

**Published:** 2025-11-19

**Authors:** Wioletta Tomaszewska, Emmanuel Arriaga-Varela

**Affiliations:** 1 Museum and Institute of Zoology, Polish Academy of Sciences, Twarda 51/55, 00-818 Warszawa, Poland; 2 Red de Biodiversidad y Sistemática, Instituto de Ecología, A.C., Carretera Antigua a Coatepec 351, El Haya, Xalapa 91070, Mexico; arriagavarelae@gmail.com

**Keywords:** Coccinelloidea, handsome fungus beetles, ‘merophysiine complex’, phylogenetic relationships, south-eastern Palaearctic, taxonomy, China

## Abstract

A new genus and species of the family Endomychidae (Coleoptera: Coccinelloidea), *Hadroscelis sinensis* Tomaszewska and Arriaga-Varela **gen. et sp. nov**., were described, diagnosed, and illustrated from the south-eastern Palaearctic. In order to test the systematic placement of the new taxon within the subfamily Danascelinae and of Danascelinae within Endomychidae, a phylogenetic analysis was conducted. A dataset of 35 morphological characters scored for 16 species, members of each Endomychidae subfamily and a representative of Coccinellidae as a distant outgroup, was analyzed using maximum parsimony. The results of the analysis unequivocally indicated *Hadroscelis* as a new, monotypic genus and the most basal taxon within Danascelinae. At the same time, this study formally confirmed, for the first time, the subfamily Danascelinae as a member and the basal clade of the monophyletic ‘merophysiine complex’ of the Endomychidae.

## Simple Summary

The subfamily Danascelinae of Endomychidae (handsome fungus beetles) is a group of poorly known organisms. Only two genera and three species are known from the USA, Canada, and Pakistan. We discovered a specimen from Eastern China with a combination of morphological characters that justifies the recognition of a new species and a new genus to accommodate it. We described in detail its characteristics and illustrated them. Through careful examination of the morphology of this species, we investigated its possible relationships with other Endomychidae and Danascelinae. Now we have evidence that the Danascelinae are closely related to a group within Endomychidae called ’merophysiine complex‘. Additionally, we discussed the biogeography of this subfamily and summarized that the origin of danasceline beetles could probably be traced back over 100 million years into the past, in the middle of the Cretaceous period.

## 1. Introduction

The family Endomychidae (handsome fungus beetles) in a modern sense contains nine subfamilies [1]. With about 1600 species described in about 90 genera, distributed largely in tropical areas [2], it is considered a moderately diverse and heterogeneous group of mycetophagous beetles. Nine subfamilies classified currently in Endomychidae are placed in two main clades [1]. The first clade, the ‘merophysiine complex’, is defined morphologically by adults having simple tarsi, concealed mesotrochantin, and sexually dimorphic antennae, along with a larval head with lyriform frontal arms of the epicranial suture and at most three pairs of stemmata; this group includes the subfamilies Pleganophorinae, Leiestinae, and Merophysiinae, some of the basal lineages of the family, according to Tomaszewska [3]. The second clade, the ‘endomychine complex’, is defined by pseudotrimerous tarsi in adults and a larval head with V- or U-shaped frontal arms of the epicranial suture and four pairs of stemmata. This clade corresponds to the ‘higher Endomychidae’ of Tomaszewska [3] and includes the subfamilies Endomychinae, Cyclotominae, Epipocinae, and Lycoperdininae [1].

However, the relationships of two small endomychid subfamilies, represented by Holarctic fauna, Danascelinae and Xenomycetinae, with the rest of the handsome fungus beetles remained unclear. Danascelinae, with simple linear tarsi and the larva unknown, and Xenomycetinae, with tarsi somewhat intermediate between simple and pseudotrimerous and larvae similar to the Leiestinae [4], were recovered both as sister taxa and the sister group to ‘higher Endomychidae’ in the analysis of Tomaszewska [3]. But due to the unavailability of the material, representatives of both subfamilies were not included in the study by Robertson et al. [1].

Danascelinae, established by Tomaszewska [5], currently includes two genera: monotypic *Danascelis*, proposed for *Danascelis elongata* Tomaszewska, 1999 [6] from Pakistan, and *Hadromychus*, described by Bousquet and Leschen [7] from eastern USA and Canada for *H. chandleri* Bousquet and Leschen, 2002, which also includes *H. lawrencei* Arriaga-Varela et al., 2025 [8], recently described from the western part of the USA and Canada.

In the present paper, we describe and illustrate a new genus and species, *Hadroscelis sinensis* **gen**. et **sp. nov**., from the south-eastern Palaearctic (south-eastern China), which shares diagnostic characters with both already known genera. To resolve the phylogenetic placement of the new taxon within Danascelinae and the placement of the subfamily, with its increasing discovery of species diversity, within Endomychidae, we performed a cladistic analysis.

Although the phylogeny and classification of the family Endomychidae require further morphological and molecular research, the present contribution gradually brings us closer to a more complete picture of the phylogeny and evolution of this group of beetles.

## 2. Materials and Methods

### 2.1. Morphological Material–Preparation and Processing

The newly examined specimen (=holotype) was obtained from Naturkundemuseum Erfurt, Germany (NME), and during the study, it was stored at the Museum and Institute of Zoology, PAS in Warsaw. Label information of the studied material is transcribed verbatim. Genitalia were dissected, cleared in 10% KOH solution, rinsed with distilled water, and then transferred to glycerol and examined on slides. After examination, the genitalia were transferred to a card and pinned beneath the specimen. Measurements were made using an ocular micrometre attached to an Olympus SZX16 dissecting microscope (Tokyo, Japan). The following measurements were taken: total body length, from apical margin of clypeus to apex of elytra; body width, across both elytra in the widest part; pronotal length, from the middle of anterior margin to margin of basal foramen; pronotal width, across the widest part; elytral length, along suture including scutellum. Scanning electron micrographs were taken using a HITACHI S-3400N microscope (Tokyo, Japan) under low vacuum conditions in the Electron Microscopy Laboratory at the Museum and Institute of Zoology, PAS, in Warsaw. Habitus colour photographs were taken with a 4K ultra-high-accuracy microscope, VHX-7000 Series by KEYENCE (Osaka, Japan), in the MIZ PAS, and subsequently modified in Adobe Photoshop CS6. The photographs of the male genitalia and terminalia were taken using an Olympus DP23 digital camera attached to an Olympus BX43F compound microscope; final images were produced using Helicon Focus 5.0X64 and Adobe Photoshop CS6 software. The morphological terminology follows Tomaszewska [9] and Lawrence et al. [10]. The description of the aedeagus is based on its resting position within the abdomen.

Taxonomic acts established in the present work have been registered in ZooBank (see below), together with the electronic publication

LSID: urn:lsid:zoobank.org:pub:8CB27EC9-4D48-4381-B97D-BA88159BA833

***Hadroscelis* gen. nov.**

*LSID*. urn:lsid:zoobank.org:act:88816836-EDB5-453F-8233-B582D4E05B14

***Hadroscelis sinensis* sp. nov.**

*LSID*. urn:lsid:zoobank.org:act:53DEE390-8409-42E7-87B1-B596A73FF6F8.

### 2.2. Morphological Dataset Preparation and Analysis

The morphological dataset was focused on subfamilies of the family Endomychidae (*sensu* Robertson et al. [1]). Members of the ‘merophysiine complex’ were sampled as the ingroup: two species of each of three subfamilies: Merophysiinae, Leiestinae, and Pleganophorinae. All species of Danascelinae (based on their simple tarsi) and one species of monogeneric Xenomycetinae (based on sister relationships of *Xenomycetes* Horn, 1880 with *Danascelis* indicated in previous studies, e.g., [3,11]) were used in the matrix. Representatives of the ‘endomychine complex’ of the Endomychidae—one species from the subfamilies: Endomychinae, Cyclotominae, Epipocinae, Lycoperdininae—were included as closely related outgroups. A member of Coccinellidae (*Coccinella septempunctata* (Linnaeus, 1758)) was used as a more distant outgroup. The type species for the genera were used when specimens were available for study. The characters used in the analysis were based mainly on Tomaszewska et al. [12] and Arriaga-Varela et al. [13,14] (with references to Tomaszewska [3,5]), with modifications made to provide resolution within Danascelinae and between this subfamily and potentially related taxa.

Our morphological data matrix included 16 taxa scored for 35 multistate characters. Unknown and/or inapplicable character states were scored as missing ‘?’. The list of morphological characters and the full data matrix can be found in Appendix A and Appendix B, respectively.

The maximum parsimony method (MP) was used for the phylogenetic analysis of our data matrix. The analysis was conducted in TNT (ver. 1.5, see https://www.lillo.org.ar/phylogeny/tnt/, (accessed on 15 February 2023); [15]) in order to find the most parsimonious trees (MPTs). We used the Traditional Search (TS) option with the following parameters: memory set to hold 1,000,000 trees; tree bisection–reconnection (TBR) branch-swapping algorithm with 1000 replications, saving 100 trees per replicate; and zero-length branches collapsed after the search. The characters were treated as unordered, and analysis was first performed under equal weights. Subsequently, the implied weighting option was used to reduce the effects of homoplasy. Other settings were unchanged. The analysis set with the concavity constant *K* = 3 resulted in a single tree. Character mapping was performed in Winclada (ver. 1.00.08, see www.diversityoflife.org/winclada, (accessed on 10 December 2009) [16]) with unambiguous optimization.

## 3. Results

### 3.1. Phylogenetic Analysis of Morphological Dataset

The maximum parsimony analysis (MP) under equal weights in Traditional Search (MP TS) resulted in 3 most parsimonious trees (MPTs), of the minimum length (L) = 74 steps, the consistency index (CI) = 58 and retention index (RI) = 72. Strict consensus tree calculated from these 3 MPTs has the following parameters: L = 86 steps; CI = 50; RI = 62 (Appendix C).

**Figure 1:**
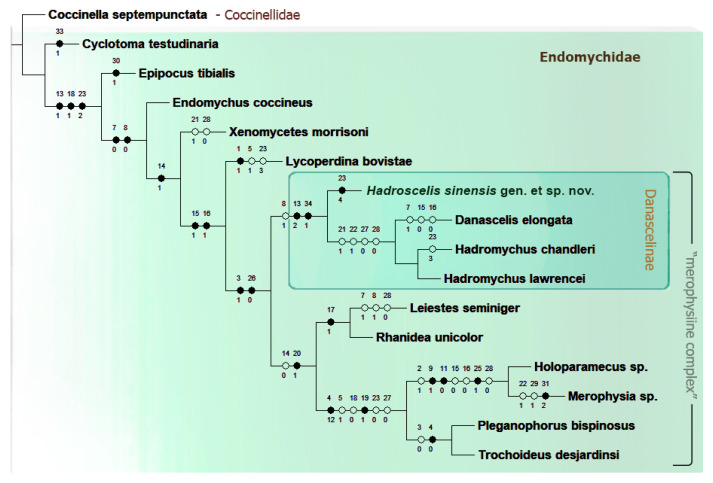
Single maximum parsimony tree based on the analysis of morphological data matrix, obtained in TNT.

The MP analysis under implied weighting (IW) in TS option, for *K = 3* resulted in a single most parsimonious tree (MPT) of the length (L) of 75 steps, consistency index (CI) = 57, and retention index (RI) = 71 (Figure 1), with the relationships between subfamilies much better resolved than in the analysis under equal weights. Therefore, this is treated as our preferred tree and our discussion and conclusions are based on this tree (Figure 1).

Our preferred tree (Figure 1) displays all lineages recognized currently as Endomychidae subfamilies. However, the ‘endomychine complex’ was not recovered as a distinct clade. Instead, each single subfamily serves as a sister group to all remaining subfamilies used in the analysis. This is, most probably, a result of limited taxon sampling of members of this group, which was playing the role of closely related outgroup for ‘merophysiine complex’, the main subject of the present research. Contrary to ‘endomychine complex’, ‘merophysine complex’ is recovered as a monophyletic group expanded by Danascelinae in a sister group position to Leiestinae + (Merophysiinae + Pleganophorinae), and this clade is well supported by two uncontroverted synapomorphies: #3(1)-antennal sockets not visible from above; and #26(0)-penultimate mesotarsomere not or slightly reduced and not enclosed within lobe of antepenultimate tarsomere. It is worth emphasizing that Xenomycetinae are recovered in our analysis as one of the branches among the subfamilies of the ‘endomychine complex’.

Regarding the sister-group relationships between three subfamilies of ‘merophysiine complex’ of Robertson et al. [1], our morphology-based analysis revealed Leiestinae + (Merophysiinae + Pleganophorinae), instead of Pleganophorinae + (Leiestinae + Merophysiinae) as in molecular-based analysis of Robertson et al. [1]. Apart from several homoplasious charcters supporting the sister relationship of Pleganophorinae and Merophysiinae, there are two uncontroverted synapomorphies uniting these subfamilies into a clade: antenna composed of less than 11 antennomeres-#4(0,1,2); and mesocoxal cavity closed outwardly-#19(1).

The relationships within the Danascelinae clade are resolved as ((*Hadroscelis* gen. nov. + (*Danascelis* + *Hadromychus*)) (Figure 1), and this is consistent with the result obtained from the analysis under equal weights (Appendix C). The sister relationships between *Danascelis* and *Hadromychus* are based on the following homoplasious character states: #21(1)-elytral punctation more or less seriate; #22(1)-metaventrite with discrimen absent; #27(0)-trochantero-femoral attachment oblique; and #28(0)-abdomen with 5 ventrites. *Hadroscelis* characterized by its autapomorphy: #23(4)-four pairs of metaventral postcoxal pits is recovered as sister group to *Danascelis* + *Hadromychus* and as part of Danascelinae subfamily, based on two uncontroverted synapomorphies: #13(2)-pronotal base with double modifications on each side; and #34(1)-antennomere 9 in male enlarged, bulbous; and by one homoplastic character state: #8(1)-right mandible with apical tooth only (1).

Based on the results of our analysis, the new genus of Danascelinae is proposed here as fully justified.

### 3.2. Taxonomy

Order Coleoptera Linnaeus, 1758

Suborder Polyphaga Emery, 1886

Superfamily Coccinelloidea Latreille, 1807

Family Endomychidae Leach, 1815

Subfamily Danascelinae Tomaszewska, 2000

**Figure 2:**
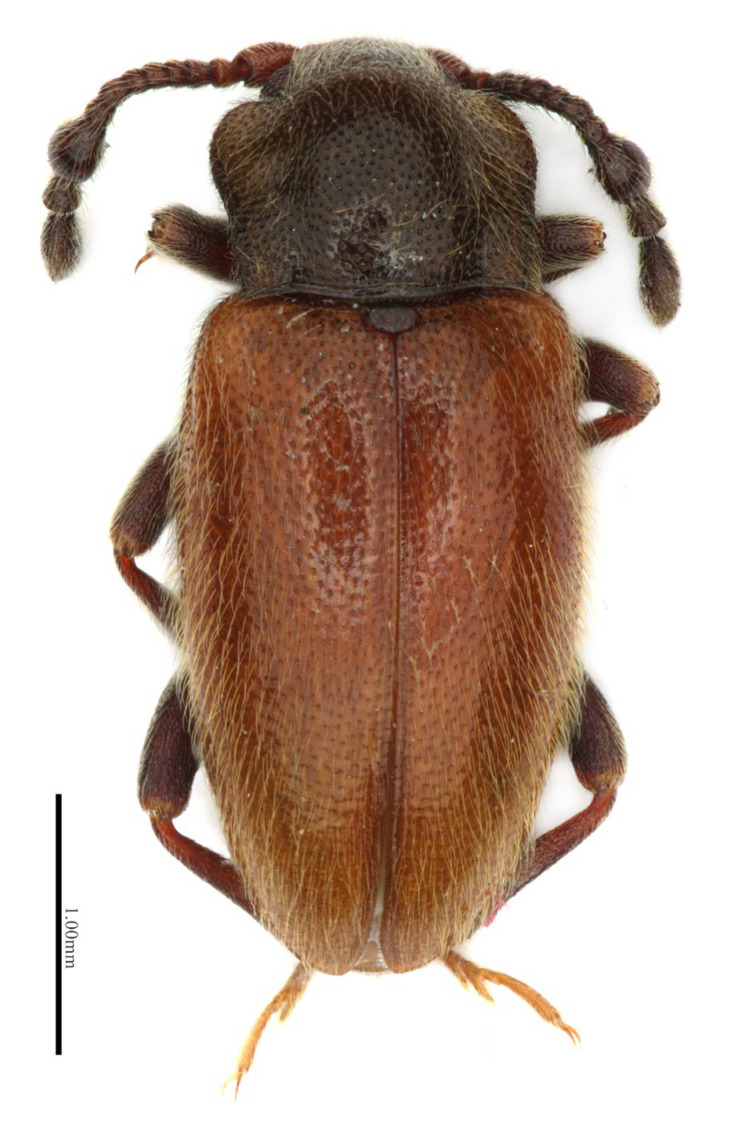
*Hadroscelis sinensis* gen. et sp. nov., holotype, male. Dorsal habitus.

**Figure 3:**
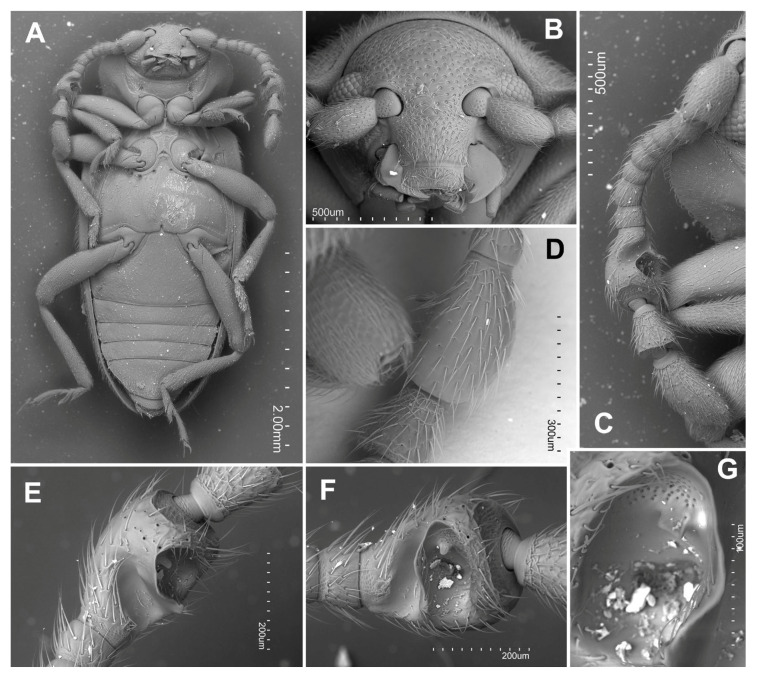
*Hadroscelis sinensis* gen. et sp. nov., holotype, male. (**A**) habitus, ventral. (**B**) head, frontal. (**C**) antenna, ventral. (**D**) ninth antennomere, dorsal. (**E**) ninth antennomere, ventro-internal. (**F**) ninth antennomere, internal. (**G**) ninth antennomere, detail of cavity.

**Figure 4:**
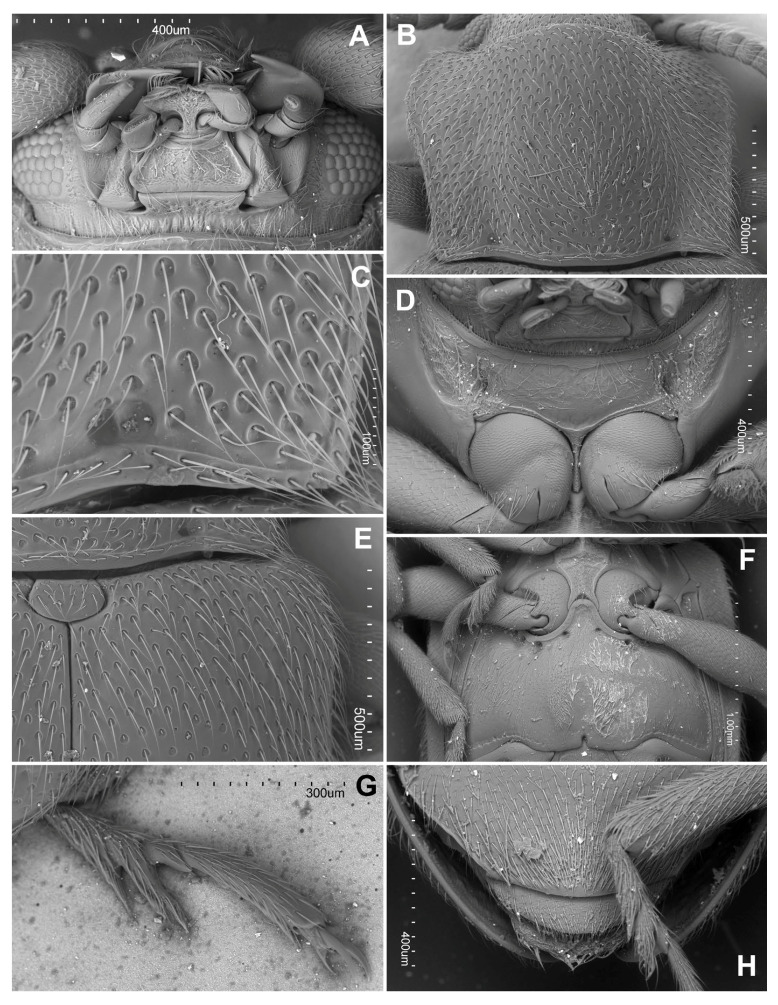
*Hadroscelis sinensis* gen. et sp. nov., holotype, male. (**A**) head, ventral. (**B**) pronotum. (**C**) postero-basal corner of pronotum. (**D**) prothorax, ventral. (**E**) scutellar shield, and anterior part of right elytron, dorsal. (**F**) meso- and metaventrite. (**G**) metatarsus. (**H**) apex of abdomen, ventral.

**Figure 5:**
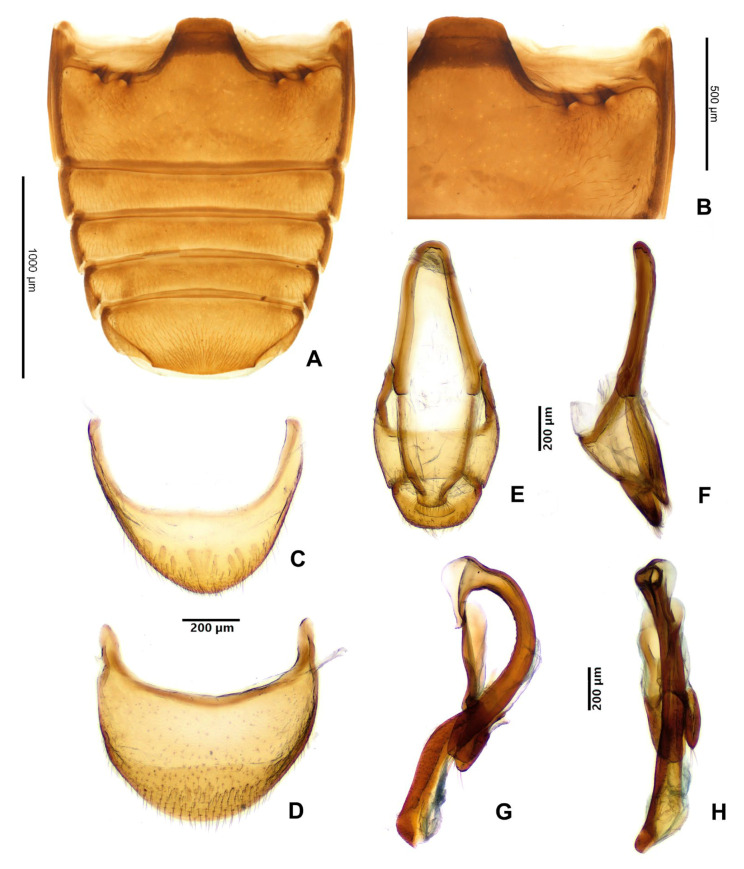
*Hadroscelis sinensis* gen. et sp. nov., holotype, male. (**A**) abdominal ventrites. (**B**) anterolateral corner of first abdominal ventrite showing the setose pores. (**C**) ventrite 6. (**D**) tergite VIII, dorsal. (**E**) male genital segment, ventral. (**F**) male genital segment, lateral. (**G**) aedeagus, dorsal view. (**H**) aedeagus, lateral.

***Hadroscelis* gen. nov.** (Figure 2, Figure 3, Figure 4 and Figure 5)

*LSID:* urn:lsid:zoobank.org:pub:8CB27EC9-4D48-4381-B97D-BA88159BA833

**Derivation of name.** The name of the new genus is a combination of letters from the names of the genera *Danascelis* and *Hadromychus*, and refers to the fact that the diagnostic characters of the new genus are a mixture of both already described genera. The gender is feminine.

**Composition.** The new genus is monotypic, represented by the type species only.

**Type species.** *Hadroscelis sinensis* sp. nov.

**Diagnosis.** *Hadroscelis* is similar to both Danascelinae genera known to this date, *Danascelis* and *Hadromychus*, in having long-oval body, and sharing characters diagnostic for the subfamily (e.g., antennomere 9 in male expanded, bulbous; pronotum with basal groove and a pair of foveae/modifications on both sides; pro-, meso, metaventrite and abdominal ventrite 1 with setose pits). Moreover *Hadroscelis* shares with *Hadromychus* left mandible provided with apical and subapical tooth (only apical tooth present in *Danascelis*), prosternal process very narrow with procoxae nearly contiguous, not prolonged to posterior margin of procoxa (prosternal process narrow but with procoxae distinctly separated, prolonged to posterior margin of coxae in *Danascelis*) and hind wings well developed (wings strongly reduced in *Danascelis*); and with *Danascelis* the new genus shares right mandible having only apical tooth (vs. apical and subapical tooth in *Hadromychus*), and antennomere 9 in male provided with deep concavity/ies on inner margin (antennomere 9 in male without apparent concavities in *Hadromychus*). *Hadroscelis, however*, can be separated from its congeners by having the pronotal base with pairs of foveae/modifications composed of a pore/pit and a concavity (not perforated) (vs. a pairs of pores in *Hadromychus* and *Danascelis*), elytral punctation irregular (vs. punctations more or less striate in *Hadromychus* and *Danascelis*), trochantero-femoral attachment heteromeroid (vs. oblique in *Hadromychus* and *Danascelis*), tarsomere 2 narrowly but distinctly lobed (slightly lobed in *Hadromychus*, hardly lobed in *Danascelis*), metaventrite with discrimen distinct, reaching nearly half-length of metaventrite (discrimen not impressed to absent in two other genera), metaventrite with 4 pairs of pits (vs. 2 pits in *Danascelis*, 2 or 3 pits in *Hadromychus*), and abdomen with ventrite 6 at least partly visible (vs. 5 ventrites in both other genera).

***Hadroscelis sinensis* sp. nov.** (Figure 2, Figure 3, Figure 4 and Figure 5)

*LSID*. urn:lsid:zoobank.org:act:53DEE390-8409-42E7-87B1-B596A73FF6F8

**Derivation of the name.** The species name refers to China, the country where the holotype was found and collected.

**Holotype. China,** male, “China, Zhejiang, Gutianshan NNR, SW10, 2010, CSP11, 647 m, 118.14 E 29.27 N, local collector/Collection Naturkunde Museum Erfurt” (NME).

**Diagnosis**. As stated for the new genus.

**Description.** Body (Figure 2 and Figure 3) length 3.85 mm, shape elongate oval, almost subparallel-sided along middle part of the elytra, moderately convex; body blackish-brown with orange elytra, tibiae reddish-brown and yellowish tarsi, weakly shiny, covered with dense, short decumbent pale yellow setae.

Head somewhat wider than long (Figure 3B) with frontoclypeal suture present; vertex without stridulatory file; eyes oval moderately large and prominent, coarsely faceted, with nearly 90 facets; interfacetal setae absent. Antennal insertions mostly hidden in dorsal view. Antenna slightly longer than head and pronotum combined, composed of 11 antennomeres (Figure 3C); antennomeres 2, 3, 5, 7 and 8 about as long as wide, antennomeres 4 and 6 weakly wider than long, club formed by three antennomeres, most probably sexually dimorphic: in males antennomere 9 bulbous, much wider than antennomeres 10 or 11 (Figure 3D), ventrally with two large excavation (Figure 3E,F), anterior concavity deeper with a patch of pores anteriorly (Figure 3F); antennomere 10 weakly wider than long, trapezoidal, widest anteriorly, and antennomere 11 oblong-oval, twice as long as antennomere 10, rounded apically. Mandibles with single apical tooth on right one, with apical and subapical teeth on left one; outer edge not denticulate. Maxilla with 3-segmented palpi (Figure 4A); palpomere 1 moderately elongate, 2 very short, transverse, terminal palpomere elongate and comparatively narrow, truncate at apex with oval field of sensilla. Labium (Figure 4A) 3-segmented with palpi separated by about width of palpiger; terminal palpomere longer than penultimate, rather flat and almost parallel sided, conical with apex truncate and with large oval field with sensilla. Mentum trapezoidal (Figure 4A), about 2.1 times wider near base than long, edges marginated by elevated carinae, without longitudinal medial ridge. Tentorium not studied.

Prothorax. Pronotum transverse (Figure 4B), 1.35 times wider than long, sides weakly sinuate and widest near anterior third; pronotal disc weakly convex, lateral areas weakly explanate; anterior angles weakly protruding forward, roundly blunt; posterior angles almost right-angled; lateral edges narrowly bordered; anterior edge without distinct margin, posterior edge with complete moderately broad margin, base of pronotum with a comparatively shallow concavity and a smaller, deeper pit, on each side, longitudinal sulci absent (Figure 4C). Hypomeral area with weak concavities for fore femora. Prosternum in front of coxa shorter than longitudinal procoxal diameter, with a small setose pit in front of each coxa (Figure 4D); prosternal process narrowed at mid-length, not prolonged to posterior margin of coxa, coxae almost contiguous; procoxal cavities externally open.

Pterothorax. Scutellar shield small, about twice as wide as long, broadly rounded posteriorly (Figure 4E). Elytra 2.55 mm long, 1.6 times as long as wide, about 2.9 times as long and 1.25 as wide as pronotum, wider near anterior fourth, convergent to rounded apex; sutural stria rather weakly marked, present along apical half of each elytron. Punctation (Figure 4E) composed of large foveolate setiferous punctures not forming striae separate by each other by 1.5–2.5 diameters, and a few sparse smaller simple setiferous punctures. Mesoventrite with very week raised longitudinal median carina, and more conspicuous longitudinal carinae demarcating median third (Figure 4F), procoxal rests with hardly defined fossae; anterior margin with setose pit near each anterolateral angle. Mesocoxal cavities open laterally (=mesepimeron reaching mesocoxal cavity), mesotrochantin exposed. Metaventrite slightly narrowing anteriorly, with metaventral process subtriangular with rounded apex (Figure 4F); with anterior margin distinctly and homogeneously bordered, with three pairs of large pits of subequal diameter below the mesocoxae, and with a pair of smaller pits near base of metacoxal process; lateral margins with bordering narrow and shallow, crenulate at basal third, posterior margin with bordering continuous with lateral ones but wider, crenulate, not distinct at center; discrimen distinct at basal half, marked at base as deep notch of the posterior margin (Figure 4F); posterior half of metaventrite with a pair of patches of dense, short setae on each side of mid-line. Metanepisternum with setose pit near anterior margin (Figure 4F). Hind wings well developed, with anal lobe present.

Legs. Trochantero-femoral attachment heteromeroid (Figure 4F); femur subcylindrical, slightly flattened, wider in distal half, with weak cavity along inner margin to receive tibia; tibiae lacking apical spurs. Tarsal formula 4–4–4 in both sexes (Figure 4G,H); tarsomeres 1 and 2 short, ventrally projected distally, tarsomere 2 weakly lobed laterally; tarsomere 3 shorter but well visible in ventral view; tarsomere 4 almost as long as previous three tarsomeres combined; tarsal claw without tooth; empodium bisetose.

Abdomen with six ventrites (Figure 4H and Figure 5A,C). Ventrite 1 with a pair of large setose pit below each metacoxal (Figure 5B); about as long at midline as ventrites 2–5 together; ventrite 6 arcuate apically (Figure 4H). Tergite VIII with margin rounded (Figure 5D).

Male terminalia and genitalia. Male genital segment (Figure 5E,F) with sternite IX well developed; paired apophyses fused roundly at apex. Aedeagus (Figure 5G,H) with median lobe (=penis) sclerotized, with membranous gonopore at apex, markedly curved, somewhat in form of ‘?’ (question mark), weakly widened at base; tegmen in form of short, sclerotized ring with marked parameres provided with long setae at their apices, tegminal strut flat.

## 4. Discussion

### 4.1. Taxonomic History of Danascelinae

*Danascelis elongata* Tomaszewska, 1999, the first described genus and species of today’s endomychid subfamily Danascelinae, was originally placed in the subfamily Epipocinae [6] based on its morphological similarity to species of *Danae* Reiche, 1847 and *Tragoscelis* Strohecker, 1953, classified then in Epipocinae.

The monotypic subfamily Danascelinae was proposed as the result of the first cladistic analysis of the family Endomychidae done by Tomaszewska [5]. That analysis supported *Danascelis* as a separate clade, defined by two apomorphies: male antennomere 9 with a tuft of long setae in a deep concavity on the inner edge, and the base of pronotum with two pairs of deep, oval cavities provided with pits. However, any close relationship with Danascelinae was not recovered. The second genus of this group, *Hadromychus* Bousquet and Leschen, 2002 [7] was proposed and described as very similar to *Danascelis*. In their paper, Bousquet and Leschen [7] commented that they added *Hadromychus* in Tomaszewska’s [5] data matrix but the genus was placed in their analysis in an unresolved polytomy of Endomychidae, similar to the tree shown in Tomaszewska ([5]: fig. 843). So, the placement of *Hadromychus* was not formally fixed.

An expanded cladistic analysis of the family Endomychidae to resolve the relationships between its subfamilies, was performed by Tomaszewska in 2005 [3], based on adult and larval characters. This time, *Hadromychus* was also included in the data matrix, and according to the results of that analysis Danascelinae formed a monophyletic group supported by an adult character state: base of pronotum with paired foveae on each side. The second postulated synapomorphy for Danascelinae in the analysis of Tomaszewska [5], male antennomere 9 provided with tuft of long setae growing from deep concavity on inner edge, remained an autapomorphy for *Danascelis*, as this character was not observed in *Hadromychus* [7]. Danascelinae was recovered in that analysis as sister group to the monogeneric, Nearctic Xenomycetinae, although this relationship was supported by only one homoplastic character state: an intercoxal process of mesoventrite, possessing a distinct elongate, median ridge extending at least along anterior half-length of process (the character shared also with some Lycoperdininae). What was interesting Danascelinae + Xenomycetinae formed a sister taxon to ‘higher endomychidae’. This was, however, based on one larval character, even when the larvae of Danascelinae are unknown till present. Therefore, the taxonomic placement and sister relationships of Danascelinae remained unresolved.

This situation was, unfortunately, not changed in the groundbreaking work by Robertson et al. [1] that brought many changes to the classification of Coccinelloidea and Endomychidae, due to the lack of the Danascelinae and Xenomycetinae material for molecular analysis.

### 4.2. Systematics–Current Research and the Present Position

With a discovery of more taxonomic diversity of this group, in this study we tried to solve the existing problems. And the cladistic analysis of the morphological characters conducted by us: (1) confirmed the monophyly of the subfamily Danascelinae containing three genera including the new genus and species described here; (2) resolved the relationships within the subfamily; and (3) identified the closest relatives of Danascelinae within Endomychidae, supporting its placement in the ‘merophysiinae complex’.

The MP analysis revealed the subfamily Danascelinae as a monophyletic group including three main lineages, recognized as distinct genera, *Hadromychus, Danascelis* and the new genus proposed here, *Hadroscelis* gen. nov. The monophyly of the subfamily is morphologically well supported by two uncontroverted synapomorphies: #13(2)-pronotal base with double modifications on each side; and #34(1)-antennomere 9 in male enlarged, bulbous; and by one homoplasious character state: #8(1)-right mandible with apical tooth only (1).

The relationships between genera within Danascelinae are recovered as *Hadroscelis* + (*Danascelis* + *Hadromychus*), suggesting south-eastern Palaearctic as a place of origin of Danascelinae ancestor, and a further diversification of this group in eastern Palaearctic and Nearctic.

The sister group relationship between Palaearctic *Danascelis* and Nearctic *Hadromychus* are supported by four homoplastic character states: #21(1)-elytral punctation more or less seriate; #22(1)-metaventrite without discrimen; #27(0)-trochantero-femoral attachment oblique; and #28(0)-abdomen with 5 freely articulated ventrites.

Although the Danascelinae genera are morphologically distinct, no character state defining *Hadromychus* (with its two species) was optimized in the resulted tree. However, the detailed differential diagnosis, separating it from its congeners is provided in taxonomy section. The monotypic *Danascelis* is morphologically well-defined by four homoplastic characters: #7(1)-left mandible with apical tooth only; #15(0)-prosternal process at least 0.2 times as wide as procoxa; #16(0)-prosternal process reaching at least hind margin of procoxae; and #24(1)-hind wing reduced or absent. The monotypic, Palaearctic *Hadroscelis* is supported by a single uncontroverted synapomorphy: #23(4)-metaventral postcoxal pits (on anterior margin of metaventrite, adjacent to posterior margin of mesocoxal cavities), four pairs present.

The placement of Danascelinae within Endomychidae, as a sister group to ‘merophysiine complex’ of Robertson et al. [1] is recovered and supported morphologically by two uncontroverted synapomorphies: #3(1)-antennal sockets not visible from above (or mostly hidden) (although visible in Pleganophorinae); and #26(0)-penultimate mesotarsomere not or slightly reduced and not enclosed within lobe of antepenultimate tarsomere. This indicates Danascelinae as members of ‘merophysiine complex’ clade and an expanded ‘merophysiine complex’ is here formally proposed.

### 4.3. Biogeography

We are aware that having such limited data about the subfamily, the discussion about its biogeography is rather speculative and reliable conclusions cannot be presented at this stage. However, it may serve as working hypothesis and be useful for further study of phylogeny and evolution of Danascelinae and the family Endomychidae.

The known biogeographical pattern of Danascelinae displays clearly disjunct distribution between Nearctic and eastern Palaearctic taxa. From among species of three genera of the subfamily, two known species of *Hadromychus* are endemic to North America (Nearctic region); *Danascelis elongata* is endemic to Pakistan and *Hadromychus sinensis* is presently known from south-eastern China (both in south-eastern Palaearctic).

The species of *Danascelis* and *Hadroscelis* are currently known from the type localities only, and the species descriptions were based on few and single specimen, respectively. This may indicate that they are rare species. On the contrary, species of *Hadromychus* have rather wide distribution (one in the western, and the second in the eastern USA and Canada), and there are numerous specimens of both species known in the collections. This may suggest that present distribution of the subfamily in Palaearctic region has rather refugial or relictual character while Nearctic may be the centre of further diversification of this group.

The published dated phylogenies of Coleoptera suggest that the subfamily Coccinelloidea originated between the Late Triassic to Early Jurassic [17,18,19,20].

Endomychidae are represented in the fossil records by 19 named species reported from Meso- and Cenozoic fossil deposits [21,22]. Most of them are from Kachin, Baltic and Bitterfeld amber deposits [2,11,23,24,25,26,27,28,29]. Most of these fossil handsome fungus beetles are described from the Cretaceous amber of Myanmar and most of them belong to the ‘merophysiine complex’ (Leiestinae, Pleganophorinae and Merophysiinae), but members of the ‘higher Endomychidae’ are also known from that period [11,21]. This suggests that at least some evolutionary lineages of Coccinelloidea including Endomychidae were well established 100 million years ago (e.g., [11,25,27,28]).

The study of Hsiao and Tomaszewska [21] who investigated the diversity dynamics of Endomychidae, based on the representative fossil assemblages of the family from the Mesozoic and Cenozoic, suggested that the overall diversity of handsome fungus beetles increased during Meso-Cenozoic period, what is consistent with previous molecular phylogenetic studies (e.g., [19]), indicating a stable and moderate net diversification rate of Endomychidae since its estimated origin in the Late Jurassic.

Although nothing is known about extinct Danascelinae, its phylogenetic placement as basal lineage of the ‘merophysiine complex’, recovered in our study, indicates origin of this subfamily at least in Cretaceous and possibly further diversification during a time of easy dispersal across Laurasia, before North America separated from Eurasia in the early Cenozoic.

In the light of what was said above, the assumption about the centre of the origin and subsequent diversification of Danascelinae is very tentative, and we are not able to hypothesize whether vicariance or dispersal was the main process shaping the present distribution of this group. If it was a result of vicariance, the split between the Palaearctic and Nearctic populations should have occurred with the opening of the North Atlantic Ocean in the early Cenozoic [30].

## 5. Conclusions

▪ The results of our phylogenetic analysis, indicated unequivocally that the new species described based on the specimen collected in Zhejiang, China constitutes a new monotypic genus belonging in the subfamily Danascelinae and as the most basal lineage sister to (*Danascelis* + *Hadromychus*); and the subfamily is recovered and assigned for the first time as a member of ‘merophysine complex’, as sister to (Leiestinae + (Pleganophorinae + Merophysiinae).
▪ Danascelinae is recovered as sister-group to the subfamilies of the ‘merophysiine complex’ of Endomychidae based on characters such as second tarsomere not enclosing the third one and antennal insertions mostly invisible from above.
▪ *Hadroscelis sinensis* gen. et sp. nov. is distinguished from other Danascelinae based on the type of basal impressions on the pronotum (one excavated pit and one shallow concavity on each side), the number of setose pores on the anterior margin of the metaventrite (four pores on each side), the type of trochantero-femoral attachment (heteromeroid) and the shape of the second tarsomeres (slightly lobed).
▪ The Danascelinae might have split from other endomychids at least as far back as the Cretaceous and the split between the Palaearctic and Nearctic populations of the subfamily could have occurred in the early Cenozoic; and the currently known disjunct distribution might represent a relictual pattern or refugia.
▪ Further research is needed to understand the biogeographic pattern of Danascelinae species and the processes that led to it.

## Data Availability

The original contributions presented in this study are included in the article. Further inquiries can be directed to the corresponding author.

## Appendix A. List of Morphological Characters Used in Cladistics Analyses

1. Head with occipital file: absent (0); present (1).
2. Head with antennal grooves: short (0); long (1) absent (2).
3. Antennal sockets: well visible from above (0); not visible (mostly hidden) (1)
4. Antenna: 4-5/7 (0); 8 (1); 9-10 (2); 11 (3).
5. Antennal club: 3-segmented (0); 1 or 2-segmented (1).
6. Fronto-clypeal suture: absent (0); present (1)
7. Left mandible: with apical and subapical tooth (0); with apical tooth/teeth only (1)
8. Right mandible: with apical and subapical tooth (0); with apical tooth only (1)
9. Labial palp with palpomere 2: subcylindrical or transverse (0); oval, inflated (1).
10. Tentorium with anterior arms: separate (0); meeting medially (1).
11. Corpotentorium absent (0); present (1).
12. Pronotum with basal and/or lateral sulci/modifications: absent (0); present (1).
13. Pronotal base smooth (0); with single modification (pit/pore/concavity) laterally (1); with double modifications on each side (2).
14. Prosternal precoxal pits: absent (0); present (1).
15. Prosternal process: at least 0.2 times as wide as procoxa (0); very narrow with procoxae nearly contiguous (1).
16. Prosternal process: reaching at least hind margin of procoxae (0); short, not prolonged to posterior margin of procoxa (1).
17. Mesoventrite with intercoxal process bicarinate (boat-shaped): absent (0); present (1).
18. Mesoventral postcoxal pits: absent (0); present (1).
19. Mesocoxal cavity: open outwardly (0); narrowly closed outwardly (1)
20. Mesotrochantin: at least partially exposed (0); concealed (1).
21. Elytral punctation: irregular (0); more or less seriate (1)
22. Discrimen: present (0); absent (1)
23. Metaventral postcoxal pits (anterior margin of metaventrite, adjacent to posterior margin of mesocoxal cavities): absent (0); one pair present (1); two pairs present (2); three pairs present (3); four pairs present (4).
24. Hind wing: well developed (0); reduced or absent (1)
25. Tarsal formula: 4-4-4 (0); 3-3-3 (1).
26. Penultimate mesotarsomere: not or slightly reduced and not enclosed within lobe of antepenultimate tarsomere (0); highly reduced and partly or entirely enclosed within ventral lobe of antepenultimate tarsomere (1).
27. Trochantero-femoral attachment: oblique (0); heteromeroid (1).
28. Abdomen: with 5 freely articulated ventrites (0); with ventrite 6 visible (1)
29. Abdominal ventrite 1: without postcoxal lines (0); with postcoxal lines (1).
30. Sternite (IX) of male genital segment with lateral edges deeply, asymmetrically curved inwardly: absent (0); present (1).
31. Tegmen: without penis guide (0); with penis guide (1); tegmen absent (2).
32. Ovipositor without infundibulum (0); with infundibulum (1).
33. Ovipositor with sperm duct attached: directly to spermatheca (0); to broad connection between spermatheca and accessory gland (1).
34. Antennomere 9: simple (0); enlarged, bulbous (1).
35. Antennomere 9: without concavities (0); with deep concavity(ies) (1).
